# New features in *ATSAS-4*, a program suite for small-angle scattering data analysis

**DOI:** 10.1107/S1600576725002481

**Published:** 2025-04-15

**Authors:** D. Franke, T. Gräwert, D. I. Svergun

**Affiliations:** a BIOSAXS GmbH, Notkestrasse 85, Hamburg, 22607, Germany; Argonne National Laboratory, USA

**Keywords:** small-angle scattering, data analysis, biological macromolecules, nanostructure, structural modeling, *ATSAS*

## Abstract

The *ATSAS* release 4 series introduces substantial architectural updates and enhanced user-friendly tools for analyzing biological macromolecules and nanostructured objects. In particular, highly requested project support for graphical user interfaces (GUIs) has been implemented. Here the state of the main window of each GUI application can be saved as a project and then restored to continue or extend analyses. All GUI applications now share a common framework, enabling various plot annotations and export as figures or text for easy data exchange with other plotting applications. The main interactive data processing and interpretation program, *PRIMUS*, has been significantly expanded with new features and integrated analysis modules. A new GUI for the program *PEAK* allows for multiple fitting functions, including Gaussian, Lorentzian and pseudo-Voigt. The online chromatography analysis program *CHROMIXS* supports multiple frame selections and subtractions, along with a newly included *EFAMIX* module for resolving partially overlapping components. The new *ATSAS* application *LAUNCHER* provides user-friendly wizards to configure and run command-line applications. Wherever feasible, operations like file reading and calculations have been parallelized to take advantage of available local compute resources. Command-line interfaces of many applications have been updated, *e.g.* with renaming and unification of common options. Improved readers/writers are implemented for Protein Data Bank and macromolecular CIF (mmCIF) file handling. The program *MIXTURES* now combines the functionalities of *BILMIX*, *LIPMIX* and *MIXTURE*, offering simplified and more versatile analyses for polydisperse systems of different shapes, also with single- or multi-layered organization. Installer packages of *ATSAS* are available for the latest versions of Windows, frequently used Linux distributions and recent macOS releases, both for Intel and ARM architectures, and are free for academic use.

## Abstract 2

*ATSAS* is a comprehensive software suite for the processing, visualization, analysis and modeling of small-angle scattering data. This article describes developments in the *ATSAS-4* release series.

## 1. Introduction

Small-angle scattering (SAS) is a powerful technique widely employed in structural biology for the study of biological macromolecules in solution (proteins, nucleic acids and large complexes) and numerous polymeric and nano­structured objects, *e.g.* lipid nanoparticles. Its primary strength lies in the ability to investigate macromolecular conformational changes, flexibility and interactions under near-physiological conditions. By providing information about the overall shape, size and structural organization of molecules in solution, SAS offers critical insights not only into individual biomolecules but also into larger complexes and mixtures that may be challenging to analyze using high-resolution techniques. SAS has become a key component in integrative structural biology, where it complements data from high-resolution methods, such as X-ray crystallography and cryo-electron microscopy, to offer a more complete understanding of macromolecular structures and their dynamic behavior (Jeffries *et al.*, 2021 ▸). Consequently, much work is underway aiming at standardization, quality, reproducibility and validation of SAS data and models (Trewhella *et al.*, 2022 ▸; Trewhella, 2023 ▸).

The *ATSAS* software package is integral to the efficient processing, analysis and interpretation of small-angle X-ray scattering (SAXS) and small-angle neutron scattering data. It includes a wide array of tools for tasks such as primary data processing, *ab initio* shape reconstruction, and the hybrid analysis of monodisperse systems, mixtures, and flexible or partially disordered systems (Manalastas-Cantos *et al.*, 2021 ▸). Over the past 30 years, *ATSAS* has continuously evolved to meet the increasing complexity of SAS data and the expanding needs of the scientific community. Early versions were centered around basic operations like data reduction and simple model fitting (Konarev *et al.*, 2006 ▸), while later iterations introduced more advanced tools for hybrid modeling, integrating SAS data with atomic structures from X-ray crystallography and nuclear magnetic resonance (Petoukhov *et al.*, 2012 ▸; Franke *et al.*, 2017 ▸). The software now supports the analysis of flexibility, mixtures and time-resolved experiments, making it broadly applicable for modern SAS studies. Time-resolved experiments can also be analyzed, which, with modern equipment, may be conducted not only on high-brilliance synchrotrons but also on laboratory X-ray sources (Rasmussen *et al.*, 2023 ▸). Additionally, modules were added to support the analysis of SAS data from soft matter systems and nanostructured materials.

The first applications that eventually became part of *ATSAS*, *e.g.GNOM* (Svergun, 1992 ▸), were developed, as customary at the time, exclusively as command-line tools. Most *ATSAS* applications are still available as command-line applications only. More recent software projects like *SASView* (https://www.sasview.org), *Scatter* (https://github.com/rambor/scatterIV), *McSAS* (https://github.com/BAMresearch/McSAS3), *SAXSutilities* (Sztucki, 2021 ▸) and *BioXTAS RAW* (Hopkins, 2024 ▸) started out with graphical user interfaces (GUIs). These tools were more easily accessible by younger researchers accustomed to the GUI workflow who may consider command-line applications less intuitive. That said, Konarev *et al.* (2003 ▸) already initiated this shift by introducing a GUI for SAS applications, followed by the first full release of *ATSAS* (Konarev *et al.*, 2006 ▸). However, these GUIs were limited to Windows PCs. This limitation was addressed later by adopting a cross-platform GUI framework, extending graphical access to macOS and Linux users while also improving the usability of key tools like *PRIMUS* (Franke *et al.*, 2017 ▸). Nevertheless, the majority of *ATSAS* applications remained available only via the command line, posing a barrier for some users.

To mitigate, *ATSAS Online* (Petoukhov *et al.*, 2012 ▸) and the *ATSAS Portal* on the grid (Wassenaar *et al.*, 2012 ▸) offered web-based alternatives to traditional GUIs. Hosted on a small-scale cluster at EMBL, Hamburg Unit, *ATSAS Online* provided access to essential applications through a simple web interface. With the development of an *ATSAS Portal* for the grid, these initial computational resources were extended by integrating a large number of global compute nodes. This advancement enabled researchers to handle computationally demanding tasks, such as *ab initio* and hybrid modeling as well as ensemble analysis, on a much larger scale, without the need for a command line. However, these remote resources may be difficult to utilize by researchers working on confidential projects due to the openness of the architecture, potentially allowing access to the data and its analysis by third parties.

The *ATSAS-4* release series introduces new graphical interfaces to further improve accessibility, including enhancements to cross-platform applications like *PRIMUS* and *CHROMIXS* and the addition of new GUIs such as *PEAK* and *LAUNCHER*. New programs are included, *e.g.EFAMIX* for advanced analysis of mixtures. Numerous modules have been optimized for computational efficiency, read/write performance and interface organization.

This article exclusively focuses on the new developments in *ATSAS-4* and is not intended as an exhaustive overview of all components of the *ATSAS* package. The interested reader is referred to previous *ATSAS* publications for overviews provided at the time and the detailed publications that are available for most programs.

## 2. GUIs

Multiple authors working on different applications may differently implement similar functionalities. In previous *ATSAS* releases, for example, *CHROMIXS* and *PRIMUS* (Franke *et al.*, 2017 ▸) were built using the same GUI toolkit and plotting library, but provided significantly different interfaces. Specifically, features and workflows in one application did not seamlessly translate to the other. For *ATSAS-4*, we addressed this issue by developing a unified data model and view for SAS plots within the Qt model/view architecture (https://doc.qt.io/qt-6/model-view-programming.html). We then re-implemented all plot windows and wizards in *ATSAS* to utilize this common framework. As a result, every plot in every application now shares consistent features, allowing workflows learned in one *ATSAS* application to be applied across others. Notable features of this framework include:

(i) Parallelized reading of data files for improved performance.

(ii) Capability of plotting millions of data points simultaneously.

(iii) Full configurability of all plot aspects (axes, data series, annotations, legend).

(iv) Freely placeable horizontal/vertical lines, text annotations and images.

(v) Export options for figures in various formats (PNG, BMP, JPEG, TIFF, SVG or PDF).

(vi) Export of all plot data in current coordinates to CSV for easy import into other plotting applications.

The common data model also made it possible to introduce one of the most user-requested features across all applications: project support. Before *ATSAS-4*, GUI applications either did not retain any information from previous sessions or, at best, maintained a list of recently opened files. With project support, users can save the state of an application, *e.g.* currently open files, plot configurations *etc.*, and restore it later. For instance, in *PRIMUS*, this includes all data with their respective selections and the presently displayed graphical window configuration. However, previous analysis results, such as the radius of gyration (*R*_g_) for each data file, are not yet stored.

In addition to the plot model/view architecture, we also enhanced the wizard framework first introduced in *PRIMUS* (Franke *et al.*, 2017 ▸). Here, a wizard is a step-by-step guide that helps users to accomplish a specific task by breaking it down into smaller, manageable stages through dedicated wizard pages. Each page collects information or sets preferences, thus making complex processes more approachable and intuitive for users. Internally, we improved page independence and reusability, which streamlined the data flow across wizards. The most notable change for users is that, starting with *ATSAS-4*, wizards can execute tasks in parallel. For example, after configuring *ab initio* modeling, the wizard may run multiple *DAMMIF* (Franke & Svergun, 2009 ▸) tasks simultaneously, depending on the number of available system cores, hence significantly reducing overall runtime. Various wizards implement this feature in *PRIMUS*, *CHROMIXS* and the new *LAUNCHER* application.

### 2.1. LAUNCHER

**Figure 1:**
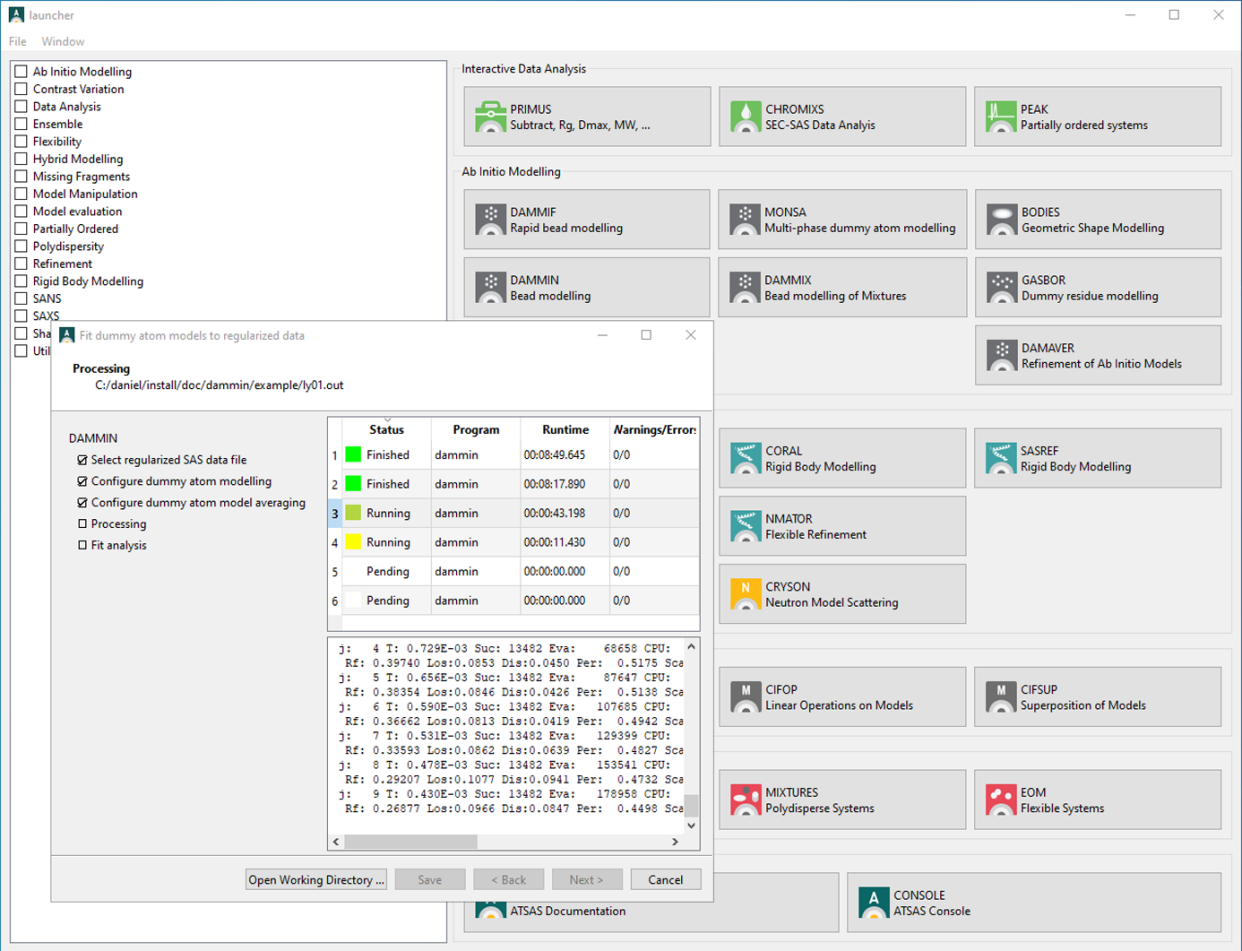
GUI of the *ATSAS* application *LAUNCHER* with a running *DAMMIF* wizard. *LAUNCHER* offers a convenient way to start other GUI applications and also a command-line console. Furthermore, it provides access to a variety of application wizards. Here the wizard for the bead modeling application *DAMMIF* (Franke & Svergun, 2009 ▸) is shown. Although six repetitions of *DAMMIF* were configured, given the hardware limitations of a virtual machine, only two are running in parallel (alternating yellow/dark yellow indicators), two are already completed (green), and two more are pending (white). It is possible to start multiple wizards in parallel; CPU resources will then be shared among them.

One common challenge for new users of *ATSAS* is navigating through the wide array of available tools and options. To address this, the ‘*ATSAS* application launcher’ was developed as a user-friendly graphical interface designed to simplify access to complex command-line applications (Fig. 1 ▸). By providing a collection of intuitive wizards, *LAUNCHER* streamlines the configuration and application of these tools, reducing the learning curve for users.

*LAUNCHER* offers wizards for the most popular tasks such as *ab initio* modeling, hybrid modeling, model manipulations, and mixture analysis and flexible systems. Each wizard operates independently, allowing users to run multiple wizards simultaneously, with all tasks managed by a shared internal queue. Much like a cluster task manager that distributes jobs across multiple nodes, *LAUNCHER* allocates system resources, running only as many tasks as available CPU cores allow to prevent the system overload. This design is functionally similar to submitting tasks through an *ATSAS Online* interface but operates locally and securely. Each wizard with three or more pages shows an outline of the required configuration, indicating the completed steps. Once the wizard tasks are submitted, the final configuration is saved to a .launcher file in the working directory. This configuration can be used as a starting point for a new task by opening it with the *LAUNCHER* application and then applying any required changes before resubmitting to the internal task queue. *LAUNCHER* is particularly beneficial *e.g.* for running multiple jobs overnight or when handling complex analyses.

In addition to the wizards, *LAUNCHER* provides quick access to other graphical *ATSAS* applications and includes a shortcut for opening a pre-configured console window, granting users a convenient way to utilize applications that are not yet available as wizards.

### 2.2. PRIMUS

*PRIMUS* (Konarev *et al.*, 2003 ▸) has been a core component of the *ATSAS* suite since its first release (Konarev *et al.*, 2006 ▸), specifically designed for the analysis of SAS data from macromolecular solutions. It remains one of the most versatile tools for SAS data analysis and, after porting to the common framework developed for *ATSAS-4*, *PRIMUS* is now capable of handling large datasets with thousands of frames and millions of data points. This makes it suitable for both single-dataset analysis and large-scale data processing tasks.

*PRIMUS* provides access to a comprehensive range of tools for basic data reduction and advanced analysis. These include modules for comparing and averaging data frames, background subtraction, and scaling and merging data interactively, allowing users to fine-tune their datasets before further analysis. The software also integrates numerous analysis wizards, enabling users to determine key structural parameters such as the *R*_g_, molecular weight (MW), maximum particle diameter (*D*_max_) and Porod volume (*V*_p_). In addition to data manipulation, *PRIMUS* supports more advanced analyses through wizards for *ab initio* shape reconstruction and hybrid modeling.

Like other GUI applications in the *ATSAS* suite, *PRIMUS* allows users to save their work in project files, preserving the state of the analysis, plot configurations and data selections.

### 2.3. CHROMIXS

**Figure 2:**
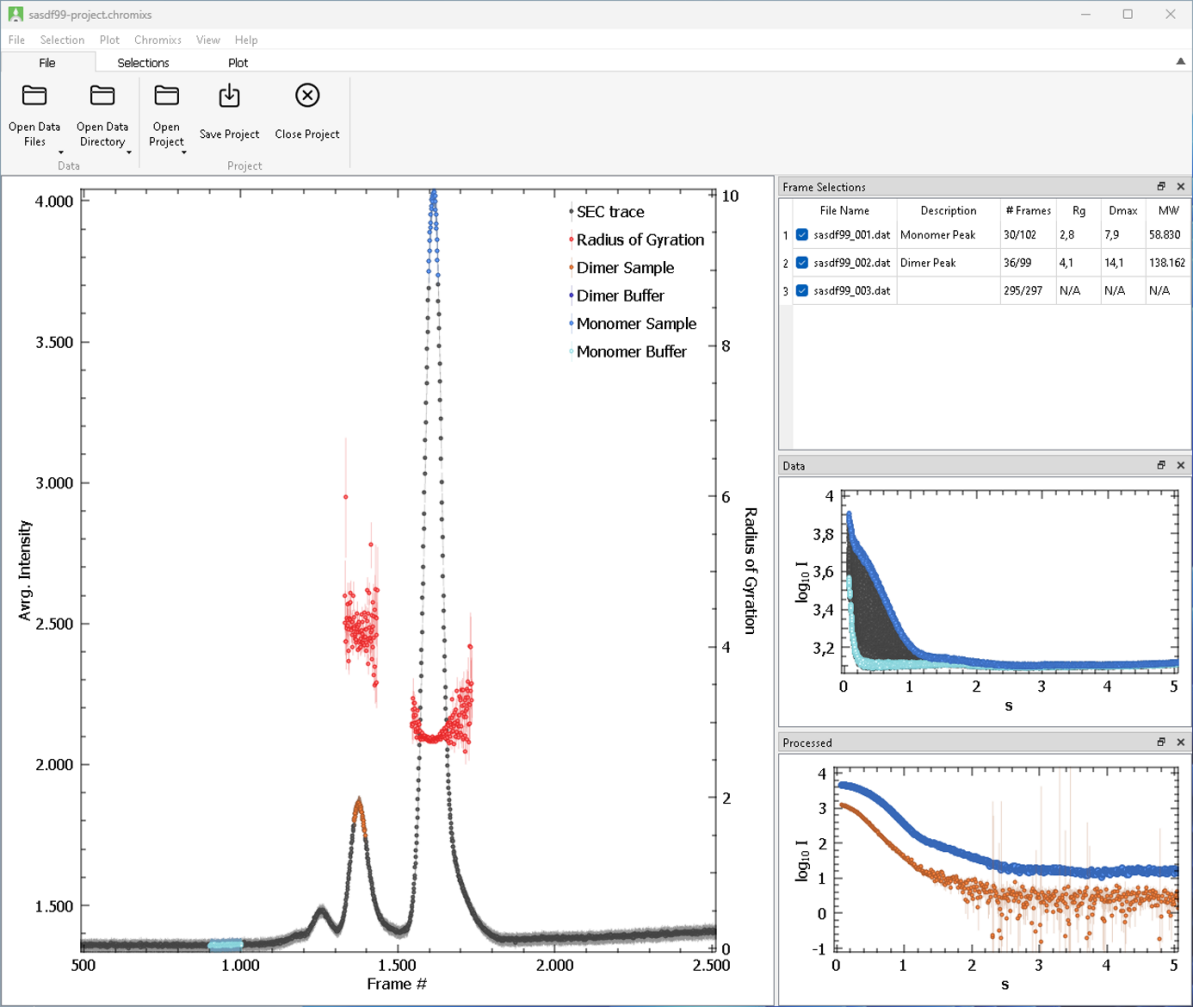
GUI for SEC-SAXS data analysis program *CHROMIXS*, here with data from the Small Angle Scattering Biological Data Bank: SASDF99 (BSA). The intensity trace together with multiple selections is shown in the left window. The top right-hand table lists the three selections: one for the monomer peak (around frame #1600), one for the dimer peak (around frame #1350) and one for the *R*_g_ calculations in a wider range (frames #1300–#1700). The plot at the center right shows all selected data frames with the current selection, here the monomer peak, shown in the corresponding colors. The plot at the lower right shows the two subtracted datasets, one for the monomer (blue), the other for the dimer (brown).

*CHROMIXS* (Panjkovich & Svergun, 2018 ▸) is designed for convenient analysis of size-exclusion or ion exchange chromatography coupled with SAXS (SEC-SAXS, IEX-SAXS) data. It automates the reduction and interpretation of large datasets generated during SEC-SAXS experiments by offering a user-friendly graphical interface that visualizes the data as averaged scattering intensity versus elution time (Fig. 2 ▸). Users may manually select the sample and buffer regions or let the program automatically detect these regions using a built-in peak-finding algorithm.

In *ATSAS-4*, besides the plot-related improvements mentioned above, *CHROMIXS* was upgraded to allow for multiple independent selections, representing different peaks or alternative selections of the same peak with varying buffer regions. This new feature, for example, allows for direct comparison of different subtractions within the interface. For each subtraction, *CHROMIXS* calculates key structural parameters, such as the *R*_g_, MW and *D*_max_, providing immediate feedback. Multi-peak systems may be further analyzed via evolving factor analysis, provided by *EFAMIX* (described below).

Opened data, frame selections and plot configuration may be saved in a project file, enabling users to resume their work later. Additionally, a ‘Package project’ feature is available, which creates a .zip archive containing all data and a corresponding project file, making it easier to share and collaborate on large datasets.

### 2.4. PEAK

**Figure 3:**
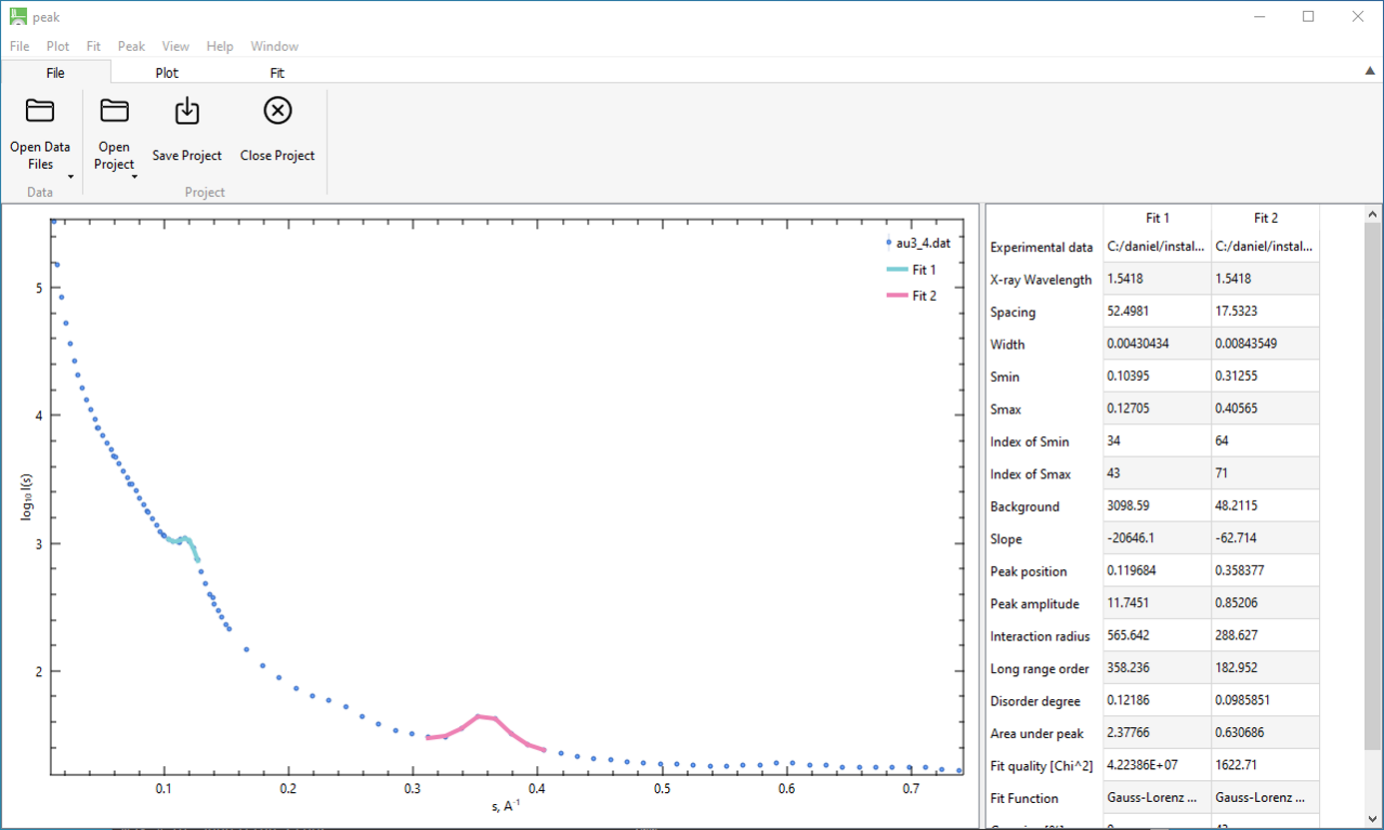
GUI of *PEAK*. Shown is the scattering from gold nanoparticles (data included in the *ATSAS* distribution package). Two peaks have been selected and the corresponding results are shown in the table.

*PEAK* (Konarev *et al.*, 2003 ▸) is designed to analyze scattering patterns of partially ordered systems. In *ATSAS-4*, the original Windows version of *PEAK* has been reimplemented and extended to use the Qt graphical software. Users interactively identify peaks in the scattering data by selecting them directly on the plot, after which the program fits the selected peaks using one of three configurable fit functions (Fig. 3 ▸): (i) Gaussian, (ii) Lorentzian or (iii) a Gaussian–Lorentzian mixture (also known as pseudo-Voigt). The software also allows for a two-parameter background subtraction to improve the accuracy of the peak fitting.

*PEAK* calculates several important structural properties from the scattering data, including *d* spacing, which corresponds to the distance between repeating units in the structure, as well as long-range order dimension *L*, interaction radius *R*_m_ and disorder level Δ/*d*. These parameters provide quantitative insights into the structural ordering in the sample. The results are visually represented as fitted curves on the plot, and all calculated values are listed in an accompanying table. When saved as a project, the software preserves the peak fits, structural parameters and plot annotations, such that the work can be seamlessly resumed as needed.

## 3. Polydisperse systems

Besides improvements in the GUIs, *ATSAS-4* also provides new and updated applications for the analysis of polydisperse systems.

### 3.1. EFAMIX

*EFAMIX* (Konarev *et al.*, 2022 ▸) is designed to work with SEC-SAXS or IEX-SAXS data. SEC-SAXS became a very popular tool in SAXS studies and multiple approaches were developed for extraction of pure component patterns from poorly resolved SEC-SAXS data (Brookes *et al.*, 2016 ▸). The *EFAMIX* program is able to analyze data from partially overlapping elution peaks, a common challenge when multiple components are not fully separated on the chromatographic column. By using evolving factor analysis (EFA) (Maeder, 1987 ▸), *EFAMIX* can restore individual scattering and concentration profiles from such mixtures, providing accurate data decomposition with minimal user intervention.

One of the core features of *EFAMIX* is its ability to handle complex, multicomponent systems. It employs a model-free approach using singular value decomposition and rotation matrices to separate overlapping scattering profiles. This method is particularly useful for biological macromolecules, which are often present in mixtures or in partially separated complexes even after chromatography purification.

Given the expected number of components, the frame range for analysis and the angular range for SAXS data processing, *EFAMIX* automatically predicts the concentration windows for each component and provides both the concentration profiles and the restored scattering curves of the components. These results can then be compared with theoretical scattering models or experimental data for further analysis.

*EFAMIX* can be used from the command line or through the ‘Evolving factor analysis’ wizard from *CHROMIXS*, based on the frame selection option of the latter.

### 3.2. MIXTURES

*MIXTURES* combines the functionality of three independent tools: *MIXTURE* (Konarev *et al.*, 2003 ▸), *LIPMIX* (Konarev *et al.*, 2021 ▸) and *BILMIX* (Konarev *et al.*, 2020 ▸). This integration enhances the analysis of complex systems composed of multiple components, such as polydisperse mixtures, lipid bilayers and vesicles, by offering a unified instrument for SAS data analysis.

The software models multicomponent systems with simple and advanced geometries. Building upon the original capabilities of *MIXTURE*, the program can handle various shapes, such as spheres, ellipsoids, cylinders and core–shell particles, incorporating parameters like size, shape and polydispersity. It also facilitates the quantitative analysis of systems where multiple scattering components coexist with each other, allowing users to account for interparticle interactions and size distributions in complex mixtures.

By incorporating tools from *LIPMIX* and *BILMIX*, *MIXTURES* enables the analysis of bilayered systems using the methodology first described by Pabst *et al.* (2000 ▸). This enables the restoration of the electron-density profiles of lipid bilayers, accounting for their polydispersity and multilamellar organization. These features are crucial for studying vesicle-based systems, such as liposomes, nanodiscs or biological membranes.

The software supports SAXS data fitting across a broad angular range, which is particularly beneficial for analyzing lipid mixtures accounting for both form factor and Bragg peak scattering portions. These analyses yield bilayer electron densities, vesicle size distributions and other structural parameters within a single modeling step, rather than relying on separate fitting procedures.

Designed with flexibility in mind, the software supports automated batch processing and parallel computations. This makes it particularly well suited for high-throughput experiments and complex datasets where simultaneous modeling of multiple components is required. This consolidation of modeling approaches into a single program offers streamlined workflows and expanded capabilities for multicomponent systems analysis within *ATSAS-4*.

Users can access *MIXTURES* through the command line or via the ‘Mixtures’ wizard in *LAUNCHER*. Wizards are also implemented for other programs dealing with mixtures and flexible systems, *e.g.OLIGOMER* (Konarev *et al.*, 2003 ▸) and *EOM* (Bernadó *et al.*, 2007 ▸; Tria *et al.*, 2015 ▸).

## 4. Technical updates

In addition to functionality expansions and improvements in GUIs, other, more technical, issues and requests from users have been addressed.

### 4.1. CRYSOL

Starting with *ATSAS 3.1*, *CRYSOL* uses the Chemical Component Dictionary (https://www.wwpdb.org/data/ccd) to determine the number of implicit hydrogens assigned to any named atom. Since the dictionary file, components.cif, is very large, parsing its contents takes several seconds – often longer than the actual scattering calculation for small mol­ecules. This slowed down *CRYSOL* computations compared with the previous implementations. As of *ATSAS 4.1*, the monolithic database is divided into individual component files, allowing *CRYSOL* to parse only the required files for a given model, thus significantly improving performance for small models.

### 4.2. Reading/writing atomic coordinates

Many applications of the *ATSAS* suite require atomic coordinates as input or provide them as output. Inputs classically come in Protein Data Bank (PDB) format or, today, in macromolecular cif (mmCIF) format (Adams *et al.*, 2019 ▸). In the 3.*x* series of *ATSAS*, read and write support for the mmCIF format was added and mmCIF was introduced as the default output format for models in all applications. Due to multiple reports of disrupted workflows caused by third-party software not yet being able to read mmCIF, we introduced the --output-format option to all relevant applications to have either PDB or mmCIF output format, so that users may select the required output format for their workflows.

In addition, PDB and mmCIF parsing was improved significantly and is now able to report on a multitude of issues, for example misaligned columns in PDB files, which used to be hard to diagnose in the past.

### 4.3. Signed packages

At the time of writing, downloading software packages from untrusted sources from the internet is discouraged; hence Windows and macOS displayed security warnings prior to running downloaded content on a system. BIOSAXS GmbH has invested in trusted certificates to sign the *ATSAS* package before its release. Signing *ATSAS* ensures that users are not unintentionally downloading malicious versions from untrustworthy sources, reducing the risk of malware infections or unauthorized access to systems. For researchers and organizations handling sensitive data, this safeguard is especially critical, as it helps maintain the security and integrity of both the software and the data processed by it.

### 4.4. *ATSAS* licenses

As of *ATSAS-4*, a license file is required to run *ATSAS* applications. Users of *ATSAS* that are affiliated with an academic or educational institute can request a free academic license from BIOSAXS GmbH by submitting an institutional email address (see https://biosaxs.com/download.php).

### 4.5. *ATSAS* at GitHub

*ATSAS* development has transitioned to Git, with all repositories now hosted on GitHub. This move enhances version control, allowing developers to track changes and collaborate more efficiently through branch management and concurrent development. GitHub further improves the process by offering secure, centralized repositories with granular access controls. It also enables continuous integration, automated testing and peer reviews, ensuring better transparency and code quality while streamlining the overall development workflow.

For registered *ATSAS* users, binaries of the latest releases are available for download from a dedicated community repository at GitHub: https://github.com/biosaxs-com/atsas-community/releases/. Users are encouraged to use the associated issue tracker (https://github.com/biosaxs-com/atsas-community/issues) for reports to the *ATSAS* developers and discussion forum (https://github.com/biosaxs-com/atsas-community/discussions) for general questions and user-to-user communication. Please note that the previously advertised (Manalastas-Cantos *et al.*, 2021 ▸) user forum at saxier.org has been closed. However, all discussions previously found at saxier.org have been imported to GitHub Discussions and are preserved there.

## 5. Conclusion

The *ATSAS-4* release series provides numerous new and enhanced features for the analysis of SAS data from biomacromolecular solutions and nanostructured systems. Many existing applications have been made more accessible and overall easier to use. The core component, *PRIMUS*, along with other GUI-based programs was rewritten in the Qt model/view architecture such that every application now shares consistent features. A new application, *LAUNCHER*, serves as a springboard to rapidly and conveniently access many *ATSAS* analysis programs, and the built-in task manager functionality helps to leverage local compute power. *CHROMIXS* now offers multiple frame selections and a wide range of options for SEC-SAXS data analysis including data processing by *EFAMIX* to handle partially overlapping elution peaks with minimal user intervention. *PEAK* provides convenient means for the analysis of partially ordered systems, while *MIXTURES* allows for comprehensive analysis of complex polydisperse systems containing multiple components. Further technical enhancements, integration and addition of new functionalities into *ATSAS* programs is under continuous development by BIOSAXS GmbH.

Licenses for *ATSAS-4* are free of charge for academic users. Installers for Windows, macOS and multiple Linux distributions are available at the *ATSAS* Community repository at GitHub: https://github.com/biosaxs-com/atsas-community/.

## References

[bb1] Adams, P. D., Afonine, P. V., Baskaran, K., Berman, H. M., Berrisford, J., Bricogne, G., Brown, D. G., Burley, S. K., Chen, M., Feng, Z., Flensburg, C., Gutmanas, A., Hoch, J. C., Ikegawa, Y., Kengaku, Y., Krissinel, E., Kurisu, G., Liang, Y., Liebschner, D., Mak, L., Markley, J. L., Moriarty, N. W., Murshudov, G. N., Noble, M., Peisach, E., Persikova, I., Poon, B. K., Sobolev, O. V., Ulrich, E. L., Velankar, S., Vonrhein, C., Westbrook, J., Wojdyr, M., Yokochi, M. & Young, J. Y. (2019). *Acta Cryst.* D**75**, 451–454. 10.1107/S2059798319004522 PMC6465986 30988261

[bb2] Bernadó, P., Mylonas, E., Petoukhov, M. V., Blackledge, M. & Svergun, D. I. (2007). *J. Am. Chem. Soc.* **129**, 5656–5664. 10.1021/ja069124n 17411046

[bb3] Brookes, E., Vachette, P., Rocco, M. & Pérez, J. (2016). *J. Appl. Cryst.* **49**, 1827–1841. 10.1107/S1600576716011201 PMC5045733 27738419

[bb4] Franke, D., Petoukhov, M. V., Konarev, P. V., Panjkovich, A., Tuukkanen, A., Mertens, H. D. T., Kikhney, A. G., Hajizadeh, N. R., Franklin, J. M., Jeffries, C. M. & Svergun, D. I. (2017). *J. Appl. Cryst.* **50**, 1212–1225. 10.1107/S1600576717007786 PMC5541357 28808438

[bb5] Franke, D. & Svergun, D. I. (2009). *J. Appl. Cryst.* **42**, 342–346. 10.1107/S0021889809000338 PMC5023043 27630371

[bb6] Hopkins, J. B. (2024). *J. Appl. Cryst.* **57**, 194–208. 10.1107/S1600576723011019 PMC10840314 38322719

[bb7] Jeffries, C. M., Ilavsky, J., Martel, A., Hinrichs, S., Meyer, A., Pedersen, J. S., Sokolova, A. V. & Svergun, D. I. (2021). *Nat. Rev. Methods Primers*, **1**, 70.

[bb8] Konarev, P. V., Graewert, M. A., Jeffries, C. M., Fukuda, M., Cheremnykh, T. A., Volkov, V. V. & Svergun, D. I. (2022). *Protein Sci.* **31**, 269–282. 10.1002/pro.4237 PMC8740826 34767272

[bb9] Konarev, P. V., Gruzinov, A. Y., Mertens, H. D. T. & Svergun, D. I. (2021). *J. Appl. Cryst.* **54**, 169–179. 10.1107/S1600576720015368 PMC7941313 33833646

[bb10] Konarev, P. V., Petoukhov, M. V., Dadinova, L. A., Fedorova, N. V., Volynsky, P. E., Svergun, D. I., Batishchev, O. V. & Shtykova, E. V. (2020). *J. Appl. Cryst.* **53**, 236–243.

[bb11] Konarev, P. V., Petoukhov, M. V., Volkov, V. V. & Svergun, D. I. (2006). *J. Appl. Cryst.* **39**, 277–286.

[bb12] Konarev, P. V., Volkov, V. V., Sokolova, A. V., Koch, M. H. J. & Svergun, D. I. (2003). *J. Appl. Cryst.* **36**, 1277–1282.

[bb13] Maeder, M. (1987). *Anal. Chem.* **59**, 527–530.

[bb14] Manalastas-Cantos, K., Konarev, P. V., Hajizadeh, N. R., Kikhney, A. G., Petoukhov, M. V., Molodenskiy, D. S., Panjkovich, A., Mertens, H. D. T., Gruzinov, A., Borges, C., Jeffries, C. M., Svergun, D. I. & Franke, D. (2021). *J. Appl. Cryst.* **54**, 343–355. 10.1107/S1600576720013412 PMC7941305 33833657

[bb15] Pabst, G., Rappolt, M., Amenitsch, H. & Laggner, P. (2000). *Phys. Rev. E*, **62**, 4000–4009. 10.1103/physreve.62.4000 11088921

[bb16] Panjkovich, A. & Svergun, D. I. (2018). *Bioinformatics*, **34**, 1944–1946. 10.1093/bioinformatics/btx846 PMC5972624 29300836

[bb17] Petoukhov, M. V., Franke, D., Shkumatov, A. V., Tria, G., Kikhney, A. G., Gajda, M., Gorba, C., Mertens, H. D. T., Konarev, P. V. & Svergun, D. I. (2012). *J. Appl. Cryst.* **45**, 342–350. 10.1107/S0021889812007662 PMC4233345 25484842

[bb18] Rasmussen, H., Nielsen, J., de Poli, A., Otzen, D. E. & Pedersen, J. S. (2023). *J. Mol. Biol.* **435**, 168194. 10.1016/j.jmb.2023.168194 37437877

[bb19] Svergun, D. I. (1992). *J. Appl. Cryst.* **25**, 495–503.

[bb20] Sztucki, M. (2021). *SAXSutilities2: a graphical user interface for processing and analysis of small-angle X-ray scattering data*, https://doi.org/10.5281/zenodo.5825707.

[bb21] Trewhella, J. (2023). *Small angle scattering*, *Part B: Methods for structural interpretation*, Vol. 678, pp. 1–22. Academic Press.

[bb22] Trewhella, J., Vachette, P., Bierma, J., Blanchet, C., Brookes, E., Chakravarthy, S., Chatzimagas, L., Cleveland, T. E., Cowieson, N., Crossett, B., Duff, A. P., Franke, D., Gabel, F., Gillilan, R. E., Graewert, M., Grishaev, A., Guss, J. M., Hammel, M., Hopkins, J., Huang, Q., Hub, J. S., Hura, G. L., Irving, T. C., Jeffries, C. M., Jeong, C., Kirby, N., Krueger, S., Martel, A., Matsui, T., Li, N., Pérez, J., Porcar, L., Prangé, T., Rajkovic, I., Rocco, M., Rosenberg, D. J., Ryan, T. M., Seifert, S., Sekiguchi, H., Svergun, D., Teixeira, S., Thureau, A., Weiss, T. M., Whitten, A. E., Wood, K. & Zuo, X. (2022). *Acta Cryst.* D**78**, 1315–1336. 10.1107/S2059798322009184 PMC9629491 36322416

[bb23] Tria, G., Mertens, H. D. T., Kachala, M. & Svergun, D. I. (2015). *IUCrJ*, **2**, 207–217. 10.1107/S205225251500202X PMC4392415 25866658

[bb24] Wassenaar, T. A., van Dijk, M., Loureiro-Ferreira, N., van der Schot, G., de Vries, S. J., Schmitz, C., van der Zwan, J., Boelens, R., Giachetti, A., Ferella, L., Rosato, A., Bertini, I., Herrmann, T., Jonker, H. R. A., Bagaria, A., Jaravine, V., Güntert, P., Schwalbe, H., Vranken, W. F., Doreleijers, J. F., Vriend, G., Vuister, G. W., Franke, D., Kikhney, A., Svergun, D. I., Fogh, R. H., Ionides, J., Laue, E. D., Spronk, C., Jurkša, S., Verlato, M., Badoer, S., Dal Pra, S., Mazzucato, M., Frizziero, E. & Bonvin, A. M. J. J. (2012). *J Grid Comput.* **10**, 743–767.

